# Pyrosequencing-based methods reveal marked inter-individual differences in oncogene mutation burden in human colorectal tumours

**DOI:** 10.1038/bjc.2011.197

**Published:** 2011-06-28

**Authors:** S Weidlich, K Walsh, D Crowther, M E Burczynski, G Feuerstein, F A Carey, R J C Steele, C R Wolf, G Miele, G Smith

**Affiliations:** 1 Biomedical Research Institute, University of Dundee, Ninewells Hospital and Medical School, Dundee DD1 9SY, UK; 2 Roche Pharmaceuticals, Nutley, NJ, USA; 3 Department of Pathology, Ninewells Hospital and Medical School, Dundee DD1 9SY, UK; 4 Centre for Academic Clinical Practice, Ninewells Hospital and Medical School, Dundee DD1 9SY, UK; 5 Cancer Research UK Molecular Pharmacology Laboratory, Ninewells Hospital and Medical School, Dundee DD1 9SY, UK

**Keywords:** K-Ras, mutation, dideoxy sequencing, pyrosequencing, colorectal tumour, personalised medicine

## Abstract

**Background:**

The epidermal growth factor receptor-targeted monoclonal antibody cetuximab (Erbitux) was recently introduced for the treatment of metastatic colorectal cancer. Treatment response is dependent on Kirsten-Ras (K-Ras) mutation status, in which the majority of patients with tumour-specific K-Ras mutations fail to respond to treatment. Mutations in the oncogenes *B-Raf* and *PIK3CA* (phosphoinositide-3-kinase) may also influence cetuximab response, highlighting the need for a sensitive, accurate and quantitative assessment of tumour mutation burden.

**Methods:**

Mutations in K-Ras, *B-Raf* and *PIK3CA* were identified by both dideoxy and quantitative pyrosequencing-based methods in a cohort of unselected colorectal tumours (*n*=102), and pyrosequencing-based mutation calls correlated with various clinico-pathological parameters.

**Results:**

The use of quantitative pyrosequencing-based methods allowed us to report a 13.7% increase in mutation burden, and to identify low-frequency (<30% mutation burden) mutations not routinely detected by dideoxy sequencing. *K-Ras* and *B-Raf* mutations were mutually exclusive and independently associated with a more advanced tumour phenotype.

**Conclusion:**

Pyrosequencing-based methods facilitate the identification of low-frequency tumour mutations and allow more accurate assessment of tumour mutation burden. Quantitative assessment of mutation burden may permit a more detailed evaluation of the role of specific tumour mutations in the pathogenesis and progression of colorectal cancer and may improve future patient selection for targeted drug therapies.

Colorectal (large bowel) cancer is the third most common cause of cancer-related death in the western world, with >36 000 new cases diagnosed annually in the United Kingdom (http://info.cancerresearchuk.org/cancerstats/types/bowel/). Despite recent advances in our understanding of disease pathogenesis and treatment, 5-year survival, particularly for patients presenting with advanced disease (Dukes’ stage C or D tumours) remains <10% (http://info.cancerresearchuk.org/cancerstats/types/bowel/survival/index.htm). Hence, there is a need for new treatment approaches and identification of optimised quantitative patient selection biomarkers for existing treatments.

Colorectal carcinogenesis is a multi-step process resulting in a progression from healthy bowel, through the formation of benign colorectal adenomas, to the development of colorectal tumours and, ultimately, to metastatic disease (Fearon and Vogelstein, 1990). Tumour formation is accompanied by an accumulation of genetic events, including chromosomal abnormalities, mutations in key tumour-suppressor genes, oncogenes and DNA mismatch repair genes, as well as epigenetic changes (Leslie *et al*, 2003; Söreide *et al*, 2006). For many years, mutations in a relatively limited number of key genes including *APC* (adenomatous polyposis coli), Kirsten-Ras (*K-Ras*) and *p53* were considered to have a central role in the development of colorectal cancer, whereas more recent data have identified an increasingly complex network of genes and mutations associated with disease pathogenesis (Fearon and Vogelstein, 1990; Smith *et al*, 2002; Leslie *et al*, 2003; Conlin *et al*, 2005; Suehiro *et al*, 2008), progression, survival and treatment response (Soong *et al*, 2000; Smith *et al*, 2002; Lièvre *et al*, 2006; Kato *et al*, 2007).

Colorectal cancer is primarily treated by surgery, followed by adjuvant, usually 5-fluorouracil (5-FU)-based, chemotherapy in patients with adverse pathology following surgical resection (Koopman and Punt, 2009; Des Guetz *et al*, 2010). However, 5-FU is effective in less than one-third of patients, and it is currently not possible to predict which patients will respond to treatment or will experience severe treatment-associated toxicities (Longley *et al*, 2003). Similar inter-patient differences in response are seen with additional chemotherapy drugs, including irinotecan and oxaliplatin (Eng, 2009) and with novel drug treatments including bevacizumab, targeted to the vascular endothelial growth factor (Van Meter and Kim, 2010) and cetuximab, a monoclonal antibody targeted to the epidermal growth factor receptor (*EGFR*) (de Castro-Carpeno *et al*, 2008; De Roock *et al*, 2008; Karapetis *et al*, 2008; Lievre *et al*, 2008; Nicolantonio *et al*, 2008; Sartore-Bianchi *et al*, 2009).

When epidermal growth factor ligands bind to the *EGFR*, they activate a signalling pathway cascade, mediated by downstream effectors of the mitogen-activated protein kinase (*MAPK*) pathway and other pathways including the phosphoinositide-3-kinase (*PIK3CA*)/*AKT* signalling pathway. These effectors (K-Ras, *B-Raf*, *ERK*, *MAPK*, *PIK3CA* and *AKT*) influence cellular proliferation, adhesion, angiogenesis, migration and survival (Wagner and Nebreda, 2009). Blocking *EGFR* with antibody-based drugs including cetuximab (Erbitux) or panitumumab (Vectibix) inhibits signalling pathways downstream of this receptor. However, mutations in the *K-Ras*, *B-Raf* or *PIK3CA* genes, common in colorectal tumours, result in structural changes in the corresponding proteins, altered effector binding and permanent activation of downstream signalling pathways, independent of *EGFR* blockade (McCubrey *et al*, 2006; Scaltriti and Baselga, 2006). Therefore, although the therapeutic benefit of *EGFR*-targeted therapy in colorectal tumours has been clearly established (Cunningham *et al*, 2004; Saltz *et al*, 2004; de Castro-Carpeno *et al*, 2008), response is preferentially observed in tumours without mutations in K-Ras, whereas patients with tumours carrying *K-Ras* mutations have response rates below 10% (Lièvre *et al*, 2006; Di Fiore *et al*, 2007; Hecht *et al*, 2007; Amado *et al*, 2008; De Roock *et al*, 2008; Karapetis *et al*, 2008; Lievre *et al*, 2008; Allegra *et al*, 2009; Bokemeyer *et al*, 2009). *K-Ras* mutations have been reported in between 25 and 37% of colorectal tumours (Smith *et al*, 2002; Yuen *et al*, 2002; Calistri *et al*, 2005; Oliveira *et al*, 2007), with mutations most commonly described in codons 12 and 13 (Bos, 1989; Smith *et al*, 2010). A similar differential response to cetuximab has recently been associated with mutations in other *EGFR*-dependent signalling molecules including *B-Raf* and *PIK3CA* (Nicolantonio *et al*, 2008; Prenen *et al*, 2009; Sartore-Bianchi *et al*, 2009). The frequency of *B-Raf* and *PIK3CA* mutations in colorectal tumours has been estimated between 10 and 17% (Davies *et al*, 2002; Smith *et al*, 2002; Yuen *et al*, 2002; Calistri *et al*, 2005; Oliveira *et al*, 2007) and between 10 and 25% (Samuels *et al*, 2004; Velho *et al*, 2005; Nosho *et al*, 2008), respectively. V600E mutations in *B-Raf* are the most prevalent and therefore the most commonly analysed mutations in colorectal tumours (Davies *et al*, 2002; Yuen *et al*, 2002), whereas exons 9 (codons 542 and 545) and 20 (codons 1023 and 1047) have been shown to harbour ∼80% of all *PIK3CA* mutations (Samuels *et al*, 2004). Mutations in K-Ras and *B-Raf* are considered mutually exclusive (Oliveira *et al*, 2007) unlike mutations in K-Ras and *PIK3CA* (Bader *et al*, 2005).

K-Ras mutation testing is now mandated by the regulatory authorities in the United States and in Europe (Allegra *et al*, 2009; van Krieken and Tol, 2009) and is routinely used as a patient selection biomarker for cetuximab prescription in colorectal cancer patients. Current mandatory K-Ras mutation testing is limited to ‘hotspot’ codons 12 and 13, although *K-Ras* mutations have also been described at codon 61, and we have recently described several additional mutations, one of which results in an alanine-to-threonine amino-acid substitution at codon 146, occurs as frequently as previously described codon 13 mutations and seems to have a similar transforming phenotype (Smith *et al*, 2010). Analysis of these additional mutations, together with a novel amplification of the *K-Ras* gene that we have described in ∼2% of colorectal tumours (Smith *et al*, 2010), would increase the K-Ras mutation burden by more than one-third, and the current K-Ras mutation testing protocols may therefore mis-classify a significant number of patients. In addition, the majority of current mutation analyses simply classify tumours as K-Ras ‘wild type’ or ‘null’, and do not therefore consider the phenotypic consequences of inter-tumour differences in mutation burden.

It is also important to note that not all patients currently classified as ‘wild type’ for K-Ras, *B-Raf* and *PIK3CA* respond to cetuximab treatment (Lièvre *et al*, 2006; Di Fiore *et al*, 2007; Hecht *et al*, 2007; De Roock *et al*, 2008; Lievre *et al*, 2008; Bokemeyer *et al*, 2009). Although there are many reasons for this, it is possible that the limited sensitivity of conventional dideoxy sequencing-based methods of mutation assessment may fail to detect low-abundance oncogene mutations. Therefore, improved sensitive and quantitative methods for assessing mutation burden are essential, particularly for the assessment of response in biomarker-defined clinical trials. To address this issue, we have developed novel quantitative pyrosequencing-based methods for the analysis of oncogene mutation burden in colorectal tumours, and demonstrated that a significant number of tumours contain mutations in key oncogenes, which were not detected by conventional dideoxy sequencing analysis.

## Materials and methods

### Patient recruitment

Unselected Caucasian patients with a histologically confirmed diagnosis of colorectal cancer (ICD-9-CM 153. 1–4, 153.6–9, 154.0 and 154.1), undergoing surgery at the Ninewells Hospital, Dundee (*n*=102, 50 women, 52 men, age range 42–93 years) were recruited by the Tayside Tissue Bank between January 2005 and April 2007. All tumour samples used in this study were selected and dissected by an experienced pathologist, and were quality controlled by frozen section to ensure that tumour cells were present in least 60% of the sample. This is the same standard currently applied to diagnostic samples used for clinical estimation of K-Ras mutation status. Patient demographics are summarised in Table 1. Written informed consent was obtained from all patients, and the study was approved by the Tayside Tissue Banks Ethics Committee, a sub-committee of the Tayside Committee on Medical Research Ethics. All tumours were classified by the Dukes’ staging system in which Dukes’ A tumours were confined to the bowel wall, Dukes’ B tumours extended locally beyond the bowel and Dukes’ C tumours involved lymph-node metastases (Dukes, 1932). Tumour pathology was additionally classified using TNM (tumour, node, metastasis) staging (Greene, 2002), and the extent of differentiation was assessed by an experienced pathologist.

### Tissue processing

Tumour samples were taken directly from the operating theatre to the pathology department, where an experienced pathologist selected tumour tissues. Samples were then snap frozen in liquid nitrogen and stored at −80°C in the Tayside Tissue Bank until further processing. Genomic DNA was isolated from each tumour sample using a Wizard SV Genomic Purification System (Promega, Southampton, UK) according to the manufacturer's instructions, and DNA concentrations were assessed using a Nanodrop spectrophotometer (Thermo Fisher Scientific, Loughborough, UK).

### Mutation detection

#### Dideoxy sequencing

Mutations in exons 1, 2 and 3 of K-Ras, including the mutation hotspot codons 12, 13, and 61, the mutation codon 146, the *B-Raf* codon 600 and exons 9 and 20 of *PIK3CA* were detected by direct sequencing. PCR amplification was performed using the primers and reaction conditions summarised in Tables A and B, Supplementary Information. Dideoxy sequencing was performed by the DNA Analysis Facility at the Ninewells Hospital, Dundee. The software 4Peaks (http://mekentosj.com) was used to visualise and analyse the DNA sequences; mutations were identified based on automated sequence calls made by the analysis software, which were not overruled by the operator to avoid potential subjectivity of assessment of mutation burden.

#### Generation of pyrosequencing standards

A set of plasmid standards was developed for each K-Ras genotype. PCR products were amplified from cell lines or from tumour tissues of known genotype (PCR and reaction conditions are summarised in Table C, Supplementary Information) and purified using a GFX PCR DNA and Gel Band Purification Kit (GE Healthcare Life Sciences, Little Chalfont, UK). Purified PCR products were then sub-cloned into the pGEMTeasy Vector System I (Promega), and transformed into JM109 high-efficiency competent cells (Promega) following the manufacturer's instructions. Single colonies were grown in Luria-Bertoni broth+100 mg l^−1^ ampicillin, and plasmids were purified using the GenElute HP Plasmid Miniprep Kit (Sigma-Aldrich, Gillingham, UK). Sequences of plasmid inserts were verified by dideoxy sequencing (DNA Sequencing Facility, University of Dundee). For each mutation tested, a set of standards was created with the following proportions of the wild-type:mutant allele 0 : 100% 5 : 95%, 10 : 90%, 25 : 75%, 50 : 50%, 75 : 25% and 100 : 0%.

#### Pyrosequencing analysis

PCR templates for pyrosequencing analysis were amplified from 10 ng gDNA (or 0.1 pg plasmid standards) using Hotstar Taq Mastermix (Qiagen, Crawley, UK) and 5 pmol of each primer in a total reaction volume of 25 *μ*l (PCR reaction and cycling conditions are summarised in Appendix 1, Supplementary Information). In all, 1 *μ*l of each PCR reaction was analysed on an Agilent 2100 Bioanalyzer (Agilent, Edinburgh, UK) using a DNA 1000 kit, and pyrosequencing was carried out on 0.15–0.5 pmol of each PCR product using the PyroMark MD System (Qiagen) following the manufacturer's instructions, with sequencing primers and assay parameters specific to each mutation (Appendix 1, Supplementary Information). Resulting pyrograms were analysed using the PyroMark MD 1.0 software in ‘AQ mode’. For each assay, duplicate pyrosequencing analysis of tumour samples was performed, and the average of these was taken to represent the identified percentage burden of the mutant allele. The cutoff value, discriminating between the mutant and wild-type sequence, was arbitrarily assigned as 10% mutant allele burden.

### Statistical analysis

Two-sided Fisher's exact tests were used to evaluate associations between tumour mutations and age, Dukes’ staging, gender and tumour location. A *P*-value <0.05 was nominally considered to be statistically significant.

## Results

### Mutation analysis

#### Dideoxy sequencing analysis

Genomic DNA was extracted from each tumour (*n*=102) and processed as described in the ‘Materials and methods’ section. Tumour DNA was then analysed by dideoxy sequencing for mutations in K-Ras exons 1, 2 and 3 (including the hotspot codons 12 and 13 (exon 1), 61 (exon 2) and codon 146 (exon 3)), *PIK3CA* (exons 9 and 20) and *B-Raf* (V600E).

*K-Ras* mutations were identified in 26.4% of tumours, and *B-Raf* and *PIK3CA* mutations in 8.8% of tumours, when automatic base calling software was used to assign mutation status (Table 2). The majority of *K-Ras* mutations were found in codon 12 (18.6%), with a smaller number in codon 13 (5.9%). Consistent with our previous analysis of K-Ras mutation burden in colorectal tumours, no mutations were found in codon 61 (Smith *et al*, 2002). In addition, a single tumour had a mutation in codon 22 (a C to A transversion substituting glutamine (CAG) for lysine (AAG)), which had been reported previously (Tsukuda *et al*, 2000), whereas a novel 3 bp in-frame insertion on the boundary of codons 14 and 15 was also found in a single tumour. Two tumours had mutations in K-Ras codon 146. The V600E *B-Raf* mutation was found in 9 tumours (8.8% of tumours analysed), whereas the majority of *PIK3CA* mutations were found in codons 542 (3.9%) and 543 (2.9%), whereas only single tumours had mutations in codons 546 and 1047. No mutations were found in *PIK3CA* codon 1023.

#### Pyrosequencing analysis

Pyrosequencing assays for each codon were optimised to include calibration curves generated from titrated proportions of the wild-type:mutant allele, derived from cloned plasmids as described in the ‘Materials and methods’ section. Assay performance was formally assessed and is summarised in Table 3. Duplicate calibration curves were constructed for each tumour panel assessment and were used to derive adjusted percentage mutation burden calls for each genotype in duplicate. Although individual assay performances indicated accuracy at mutation burdens below 10%, we chose to use a conservative cutoff of 10% as the lower limit of quantitation for the assignment of mutation status calls.

**Figure 1:**
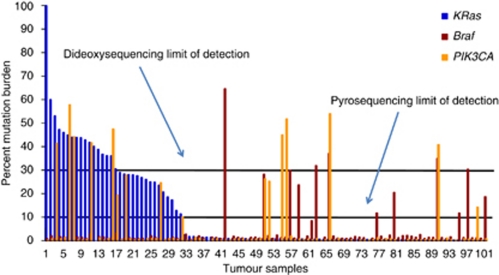
Inter-tumour variation in K-Ras, *B-Raf* and *PIK3CA* mutation burden Quantitative mutation detection was performed by pyrosequencing analysis, as described in the ‘Materials and methods’ section. Inter-tumour differences in mutation burden for K-Ras (blue), *B-Raf* (red) and *PIK3CA* (yellow) is illustrated, where each bar represents a different tumour sample. Tumours with *K-Ras* mutations are grouped to the left, with additional *B-Raf* and *PIK3CA* mutations highlighted. Arbitrary limits of detection for pyrosequencing (10% mutation burden) and dideoxy sequencing (30% mutation burden) are illustrated, highlighting the additional mutations identified by pyrosequencing analysis.

**Figure 2:**
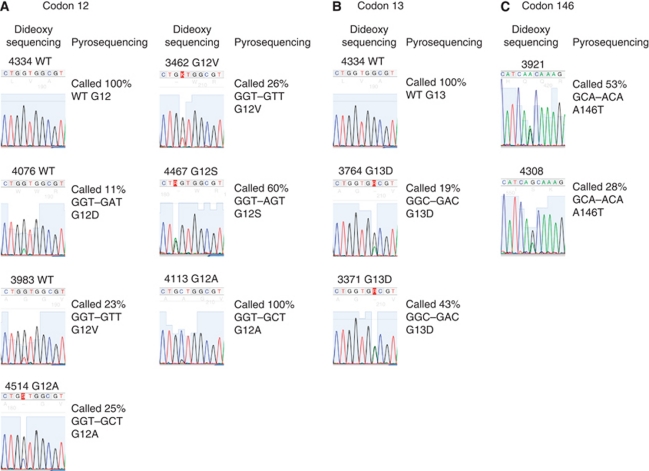
Mutation analysis of K-Ras codons 12, 13 and 146. Mutation detection was performed by dideoxy and pyrosequencing analyses, as described in the ‘Materials and methods’ section. Mutation status in samples analysed by dideoxy sequencing was assigned by automated base calling using 4Peaks software, and is shown in comparison with quantitative analysis of mutation burden, assessed by pyrosequencing. Representative analyses are illustrated.

Pyrosequencing analysis revealed mutation frequencies of 32.4, 11.5 and 13.7% for *K-Ras*, *B-Raf* and *PIK3CA*, respectively, thus significantly increasing the number of tumours with mutations in K-Ras, *B-Raf* or *PIK3CA* (Table 2). In total, an additional 14 tumours carrying *K-Ras*, *B-Raf* or *PIK3CA* mutations were identified by pyrosequencing analysis, including all mutations previously identified by dideoxy sequencing analysis. In addition, K-Ras, *B-Raf* and *PIK3CA* mutation burden ranged from 11 to 99%, 12 to 65% and 14 to 54% of cells, respectively, highlighting marked inter-tumour heterogeneity in mutation burden (Figure 1). In addition to providing quantitative assessment of mutation burden, the increased sensitivity of pyrosequencing analysis allowed us to identify low-frequency mutations (mutation burden <30%) in a subset of tumours, which had not been identified by automated base calling analysis of our dideoxy sequencing data. A comparison of K-Ras mutation calls assessed by dideoxy and pyrosequencing is illustrated in Figure 2 – similar data were obtained for *B-Raf* and *PIK3CA* (data not shown). All of the additional *K-Ras*, *B-Raf* and *PIK3CA* mutations identified by pyrosequencing analysis were retrospectively manually confirmed in the dideoxy sequencing traces (e.g., samples 3983 and 4076, Figure 2).

In confirmation of previous reports, *K-Ras* and *B-Raf* mutations were mutually exclusive in our tumour series (Yuen *et al*, 2002; Suehiro *et al*, 2008), whereas mutations in K-Ras and *PIK3CA*, in and *B-Raf* and *PIK3CA* were found together, but occurred in only 7.8 and 2.9% of tumours, respectively. A mutation in at least one of these genes, previously associated with response to *EGFR*-targeted antibody therapies, was found in 57.8% of all tumours analysed by pyrosequencing, an increase in K-Ras mutation burden of 27.4% compared with current mandatory analysis of *K-Ras* mutations restricted to codons 12 and 13, and a 33.3% increase when mutations in *B-Raf* and *PIK3CA* were additionally considered.

### Correlation with pathological data and patient details

The mutation status of K-Ras, *B-Raf* and *PIK3CA*, based on pyrosequencing assessment of mutation burden, was then correlated with patient demographics and various clinico-pathological parameters, as the increased sensitivity of pyrosequencing analysis allowed us to more accurately evaluate these correlations. Mutations in K-Ras codons 14 and 22 were excluded from this analysis, as their phenotypes have not yet been fully characterised.

**Figure 3:**
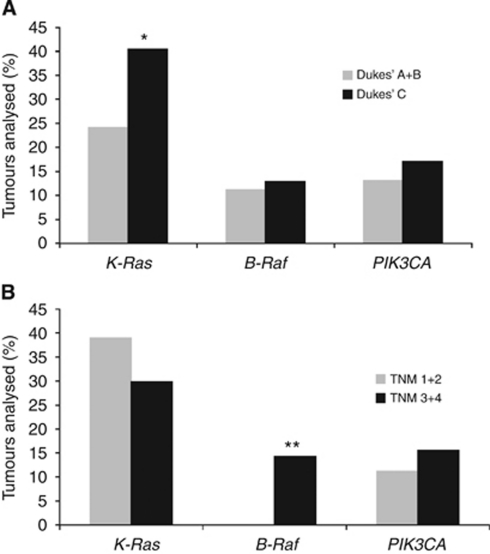
Distribution of *K-Ras*, *B-Raf* and *PIK3CA* mutations according to Dukes’ and TNM stage. The presence of mutations in K-Ras, *B-Raf*, and *PIK3CA* was determined by pyrosequencing analysis as described in the ‘Materials and methods’ section. Tumours were categorised according to (**A**) Dukes’ and (**B**) TNM staging, and further sub-divided by the presence of mutations in K-Ras, *B-Raf*, and *PIK3CA*. ^*^P=0.03, ^**^P=0.00002.

No differences in mutation frequencies comparing gender or median age were observed in our patient cohort (Table 4). In agreement with previous literature (Smith *et al*, 2002), a significantly higher proportion of rectal tumours harboured *K-Ras* mutations (40.0 *vs* 27.7%, *P*=0.04), whereas no significant differences in mutation frequencies were found for *B-Raf* (6.7 *vs* 13.9%, *P*=0.07) or *PIK3CA* (13.9 *vs* 16.7%, *P*=0.79). In addition, *B-Raf* mutation burden was significantly inversely correlated with differentiation status, in which 8.2% of moderately differentiated tumours had a *B-Raf* mutation compared with 29.4% of poorly differentiated tumours (*P*=0.0002). An additional significant correlation was found between Dukes’ stage and K-Ras mutation status, in which K-Ras mutations were more common in Dukes’ C than in Dukes’ A and B tumours (*P*=0.01) (Figure 3). This observation is consistent with our previous report (Smith *et al*, 2002), and associations between *K-Ras* mutation and poorer prognosis and time to relapse (Andreyev *et al*, 1998; Conlin *et al*, 2005). There were also significant correlations between T stage and the presence of a *B-Raf* mutation (*P*=0.00002), in which *B-Raf* mutations were restricted to more advanced tumours (T stages 3 and 4). Similarly, *K-Ras* mutations were overrepresented in tumours with lymph-node metastasis (N1 and 2) compared with lymph node-negative (N0) tumours (38.3 *vs* 25.5%, *P*=0.03).

## Discussion

Mutations in oncogenes including *K-Ras*, *B-Raf* and *PIK3CA* confer an important growth advantage to cancer cells (Vogelstein *et al*, 1988) and are found in more than one-third of all tumours. In colorectal tumours, *K-Ras* mutations have been associated with a more aggressive tumour phenotype (Smith *et al*, 2002) and reduced patient survival (Andreyev *et al*, 1998, 2001; Conlin *et al*, 2005), whereas *B-Raf* and *PIK3CA* mutations have also been associated with both disease pathogenesis and prognosis (Davies *et al*, 2002; Yuen *et al*, 2002; Calistri *et al*, 2005; McCubrey *et al*, 2006; Nicolantonio *et al*, 2008; Sartore-Bianchi *et al*, 2009; Baldus *et al*, 2010). However, the majority of current analyses of mutations in these genes are usually restricted to single amino-acid mutation hotspots, and mutation reporting is limited to a simple binary ‘wild-type’ or ‘mutant’ classification.

Several recent clinical reports provide compelling evidence that only a minority of colorectal tumours with *K-Ras*, *B-Raf* or *PIK3CA* mutations respond to novel *EGFR*-targeted monoclonal antibody therapies including cetuximab and panitumimab (Lièvre *et al*, 2006; Nicolantonio *et al*, 2008; Allegra *et al*, 2009; Sartore-Bianchi *et al*, 2009). K-Ras mutation testing is now mandatory before the prescription of these drugs, and it is therefore essential that analysis of mutation burden is as comprehensive and quantitative as possible.

We have previously described *K-Ras* mutations with previously described ‘hotspot’ codons, which significantly increase the K-Ras mutation burden in human colorectal tumours (Smith *et al*, 2010). Our current data, resulting from the analysis of an independent patient series, confirm the presence of K-Ras codon 146 mutations in colorectal tumours and report additional *K-Ras* mutations in codons 14 and 22. The codon 14 insertion, resulting in an in-frame creation of an additional glycine residue, has not previously been reported. Therefore, it is particularly interesting to note that a similar insertion mutation, K-Ras_10_Gly_11_, results in a hyperactive form of K-Ras (Bollag *et al*, 1996) – the phenotypic consequences of the codon 14 insertion are currently being evaluated in our laboratory. In contrast, the point mutation in codon 22 has been reported before (Tsukuda *et al*, 2000; Simi *et al*, 2008), although the resulting phenotype has not been fully characterised.

Importantly, our use of quantitative pyrosequencing analysis allowed us to identify mutations in K-Ras, *B-Raf* and *PIK3CA* with mutation frequencies ranging from 10 to 30%, which were not detected by automatic base calling software, routinely used in the analysis of dideoxy sequencing traces. These findings are in agreement with previous pyrosequencing studies, which have described a mutation detection threshold of 5–10% of mutant cells (Ogino *et al*, 2005; Dufort *et al*, 2009). Our data highlight an overall 13.7% increase in mutation burden (comparing the results of dideoxy and pyrosequencing analyses), and identifies a sub-set of tumours which would be erroneously classified as ‘wild type’ by conventional sequencing analysis, with potentially important implications for the prescription of *EGFR*-targeted therapies.

Although clinical response to cetuximab and related drugs is clearly dependent on K-Ras status, only one in two K-Ras ‘wild-type’ patients respond to treatment, based on current limited analysis of K-Ras mutation status (Karapetis *et al*, 2008; Lievre *et al*, 2008; De Roock *et al*, 2010). Although there are many complex factors which will inevitably contribute to variability in response, our data suggest that a significant proportion of non-responder patients may be mis-classified, either because of the presence of an additional oncogene mutation which influences cetuximab response or because of the presence of a relatively low-frequency mutation which is not detected by conventional dideoxy sequencing. Intra- and inter-tumour heterogeneity in mutation burden are also likely to be significant determinants of treatment response, and are not routinely considered in current binary ‘wild-type/mutant’ tumour classifications. In general, only a single piece of tumour is analysed for each patient, although previous studies have reported differences in mutation burden, for example, comparing tumour centres and invasion fronts (Baldus *et al*, 2010). Therefore, each individual ‘tumour’ sample may have a different normal/tumour cell ratio or a different proportion of infiltrating lymphocytes or other contaminating cell types, each of which can influence the apparent mutation burden. It is currently not possible to determine whether the marked inter-tumour variability in mutation burden observed in our patient cohort results from tumour sampling bias, or represents true differences in clonality, wherein some tumour cells contain mutations and other do not. This issue is central to the interpretation of mutation data, and we highlight the need for additional studies, for example, using laser capture micro-dissected material to address this issue. It is also clearly important that we are able to better predict the tumour phenotypes arising from varying proportions of wild-type and mutant cells – for example, should patients with 50% K-Ras mutant alleles be treated with cetuximab? In current testing protocols, these tumours would be classified as ‘mutant’, whereas a significant proportion of tumour cells have retained the ability to respond to *EGFR* inhibitors. Studies to address this issue using regulatable plasmids to vary the relative proportion of wild-type and mutant K-Ras are currently underway in our laboratory.

Recent data from our own laboratory and from that of De Roock *et al* (2010) additionally highlight the need to consider marked differences in phenotypes associated with individual oncogene mutations. Our data, in which wild-type and various mutant forms of K-Ras were transiently expressed in NIH3T3 cells, revealed significant differences in gene expression induced following the introduction of individual K-Ras mutations, suggesting that the K-Ras genotype may be a significant determinant of chemotherapy response (Smith *et al*, 2010). Consistent with this hypothesis, in a recent meta-analysis of cetuximab clinical trial data, De Roock *et al* (2010) clearly demonstrated that patients with colorectal tumours with a G13D mutation were significantly more likely to respond to cetuximab treatment than other K-Ras mutant tumours and survived longer. In additional *in vitro* experiments, these authors further demonstrated that cells expressing K-Ras G13D were phenotypically more similar to wild-type K-Ras than to cells containing alternative K-Ras mutations. These findings are consistent with previous reports highlighting differences in transforming potential, comparing K-Ras codon 12 and codon 13 mutations (Guerrero *et al*, 2000, 2002; Cespedes *et al*, 2006), and again highlights the need to extend clinical studies in this area.

Quantitative pyrosequencing-based analysis of mutation burden has also allowed us to more accurately investigate correlations between mutation burden and key clinico-pathological parameters. As would be expected from previous reports, *K-Ras* and *B-Raf* mutations were mutually exclusive in our tumour cohort, and were associated with tumours located in the rectum and colon, respectively (Yuen *et al*, 2002; Suehiro *et al*, 2008). Our data additionally confirmed our own previous report of increased K-Ras mutation burden in advanced Dukes’ C tumours (Smith *et al*, 2002) and identified a novel association associating K-Ras mutations with the presence of lymph-node metastases, consistent with the hypothesis that the *K-Ras* mutation is associated with a more aggressive tumour phenotype. Similarly, and consistent with the report of Baldus *et al* (2010), we found *B-Raf* mutation status to be inversely correlated with tumour differentiation. Like K-Ras, tumours with *B-Raf* mutations were restricted to more advanced tumours (T stages 3 and 4).

Our experimental approach has obvious application to the analysis of additional tumour types, for example, pancreatic tumours, in which *K-Ras* mutations are present in the majority (>90%) of tumours analysed (Bos, 1989), and can be easily extended to other mutation and tumour targets. In colorectal cancer, pyrosequencing-based mutation detection methods may also prove to be a powerful approach in the analysis of mutation burden in pre-malignant lesions, for example, colorectal adenomas to identify individual patients at the highest risk of disease progression and in metastatic disease for example tumours which have metastasised to liver or lymph nodes, the primary targets for adjuvant chemotherapy.

In conclusion, therefore, the use of sensitive pyrosequencing-based methods for mutation detection facilitates the identification of low-frequency tumour mutations and permits a quantitative assessment of intra- and inter-tumour differences in mutation burden. Quantitative assessment of oncogene mutation burden using pyrosequencing or other quantitative technologies including Sequenom MassArray (Sequenom, Hamburg, Germany) and next-generation sequencing methods may permit a more detailed evaluation of the role of specific tumour mutations in the pathogenesis and progression of colorectal cancer and may improve future patient selection for targeted drug therapies.

## Figures and Tables

**Table 1:** Patient demographics

	**Female**	**Male**	**Total**
No. of patients	50	52	102
Age (median (range))	74.4 (42–93)	70.3 (43–87)	72.3
			
*Dukes's stage*			
A	4 (8%)	12 (22.2%)	16 (15.7%)
B	22 (44%)	16 (30.8%)	40 (37.2%)
C	24 (48%)	24 (44.4%)	48 (47.0%)
D	0	0	0
			
*TNM stage*			
I			
T1N0MX	2 (4%)	4 (7.4%)	6 (5.9%)
T2N0MX	3 (6%)	8 (14.8%)	12 (11.8%)
II			
T3N0MX	17 (34%)	14 (26.9%)	31 (30.4%)
T4N0MX	5 (10%)	2 (3.7%)	7 (6.9%)
III			
T2N1MX	0	1 (1.9%)	1 (0.99%)
T3N1MX	6 (2%)	11 (20.3%)	17 (16.7%)
T3N2MX	10 (20%)	7 (12.9%)	17 (16.7%)
T4N1MX	4 (8%)	3 (5.6%)	7 (6.9%)
T4N2MX	3 (6%)	2 (3.7%)	5 (4.9%)
			
*Tumour localization*			
Colon	37 (74.0%)	35 (67.3%)	72 (70.6%)
Rectum	14 (26%)	17 (32.7%)	29 (29.4%)
			
*Differentiation*			
Moderate	39 (78.0%)	48 (88.5%)	87 (83.3%)
Poor	11 (22.0%)	6 (11.5%)	17 (16.7%)

Abbreviation: TNM=tumour, node, metastasis;

**Table 2:** Summary of mutation frequencies in K-Ras, *B-Raf* and *PIK3CA* as analysed by dideoxy and pyrosequencing

			**Frequency (%)**	
**Mutation**	**Nucleotide change**	**Amino-acid change**	**Dideoxy sequencing**	**Pyrosequencing**
*K-Ras*			*26.4%*	*32.4%*
Codon 12	G_34_A	Gly_12_Ser	1/102 (1%)	3/102 (2.9%)
	G_34_T	Gly_12_Cys	1/102 (1%)	4/102 (3.9%)
	G_35_T	Gly_12_Val	8/102 (7.8%)	9/102 (8.8%)
	G_35_C	Gly_12_Arg	1/102 (1%)	1/102 (1%)
	G_35_A	Gly_12_Asp	8/102 (7.8%)	8/102 (7.8%)
Codon 13	G_38_A	Gly_13_Asp	6/102 (5.9%)	6/102 (5.9%)
Codon 61	None detected	None detected	0/102	0/102
Codon 146	G_436_A	Ala_146_Thr	2/102 (1.9%)	2/102 (1.9%)
Codon 14	Ins 41–44	_14_Gly_15_	1/102 (1%)	1/102 (1%)
Codon 22	C_65_A	Gln_22_Lys	1/102 (0.6%)	1/102 (0.6%)
				
*B-Raf*			*8.8%*	*11.5%*
Codon 600	T_1798_A	Val_600_Glu	9/102 (8.8%)	12/102 (11.5%)
				
*PIK3CA*			*8.8%*	*13.70%*
Codon 542	G_1624_A	Glu_542_Lys	4/102 (3.9%)	4/102 (3.9%)
Codon 545	G_1633_A	Glu_545_Lys	3/102 (2.9%)	7/102 (6.8%)
Codon 546	A_1637_C	Gln_546_Pro	1/102 (1%)	1/102 (1%)
Codon 1023	None detected	None detected	0/102	0/102
Codon 1047	A_3140_G	His_1047_Arg	1/102 (1%)	2/102 (1.9%)

**Table 3:** Performance assessment of pyrosequencing assays

				**Assay performance**								
						**(Percentage point bias at expected allele frequency)**						
**Gene**	**Nucleotide**	**Codon**	**Substitution**	***R* ^2^**	**Slope**	**0%**	**5%**	**10%**	**25%**	**50%**	**75%**	**100%**
*BRAF*	1799	600	T>A, Val>Glu	0.998	0.918±0.0058	1.140	2.19	1.74	0.40	1.83	4.06	6.30
*KRAS*	12	34	G>T, Gly>Ser	0.997	0.956±0.0092	0.74	2.03	1.80	1.11	0.04	1.20	2.35
*KRAS*	12	34	G>A, Gly>Cys	0.997	0.930±0.0089	0	0.08	0.30	1.42	3.30	5.18	7.06
*KRAS*	12	35	G>A, Gly>Asp	0.995	0.901±0.0102	2.48	2.61	2.07	0.42	2.31	5.05	7.79
*KRAS*	12	35	G>T, Gly> Val	0.997	0.956±0.0092	0.74	2.03	1.80	1.11	0.04	1.20	2.35
*KRAS*	12	35	G>C, Gly> Ala	0.990	0.970±0.0157	0.62	4.52	4.36	3.89	3.10	2.31	1.53
*KRAS*	13	38	G>A, Gly> Asp	0.990	0.883±0.0112	0.58	0.49	0.18	2.17	5.50	8.82	12.15
*KRAS*	61	182	T>A, Lys>Gln	0.997	0.938±0.0073	3.44	1.93	1.60	0.61	1.04	2.68	4.33
*KRAS*	436	146	G>A, Ala>Thr	0.997	0.979±0.0069	0	2.1	2.65	4.4	4.35	3.45	1.65
												
*PIK3CA*	1624	542	G>A, Glu>Lys	0.992	0.972±0.0120	0	3.18	3.33	3.76	4.49	5.22	5.95
*PIK3CA*	1634	545	A>G, Glu>Gly	0.996	0.932±0.0101	0.08	2.06	1.69	0.59	1.25	3.09	4.93
*PIK3CA*	1633	545	G>A, Glu>Lys	0.994	0.949±0.0119	0.75	3.49	3.22	2.41	1.07	0.27	1.61
*PIK3CA*	1637	546	A>C, Gln>Pro	0.978	0.894±0.0217	0	1.18	1.78	3.56	6.53	9.50	12.47
*PIK3CA*	3140	1047	A>G, His>Arg	0.996	0.968±0.0084	2.15	3.83	3.66	3.16	2.33	1.49	0.66

*R*^2^, correlation co-efficient of best-fit dose–response line; Slope, slope of best–fit dose-response line.

**Table 4:** Associations between mutation status and various clinico-pathological parameters

	***N***	***K-Ras* mutant**	***B-Raf* mutant**	***PIK3CA* mutant**
Male	52	17 (32.6%)	6 (11.5%)	9 (17.3%)
Female	50	15 (30.0%)	6 (12.0%)	6 (12%)
Age median	72.4 years	71.4 years	75.3 years	72.9 years
Colon	72	**20** (**27.7%)**	10 (13.9%)	10 (13.9%)
Rectum	30	**12** (**40.0%)^§^**	2 (6.7%)	5 (16.7%)
Dukes’ A+B	54	**13** (**24.1%)**	6 (11.1%)	7 (13.0%)
Dukes’ C	47	**19** (**40.4%)^$^**	6 (12.8%)	8 (17.0%)
T stage 1+2	18	7 (38.9%)	**0**	2 (11.1%)
T stage 3+4	84	25 (29.8%)	**12** (**14.2%)^†^**	13 (15.5%)
N stage 0	55	**14** (**25.5%)**	6 (10.9%)	8 (14.5%)
N stage 1/2	47	**18** (38.3%)*	6 (12.8%)	7 (14.8%)
Moderate differentiation	85	27 (31.8%)	**7** (**8.2%)**	12 (14.1%)
Poor differentiation	17	5 (29.4%)	**5** (**29.4%)^#^**	3 (17.6%)

^§^*P*=0.04, ^$^*P*=0.01, ^†^*P*=0.00002, *P=0.03, ^#^*P*=0.0002. The (paired) bold values highlight significant results.
