# Supplementary material for: Pyrosequencing-based methods reveal marked inter-individual differences in oncogene mutation burden in human colorectal tumours
Source: Br J Cancer. 2011 Jun 28;105(2):246–54. doi: 10.1038/bjc.2011.197 (PMC3142798; doi:10.1038/bjc.2011.197)
Supplement: Supplementary Information [file bjc2011197x1.doc]

**Appendix 1 – supplementary information**

Table A: Oligonucleotide primer sequences for dideoxy sequencing analysis

Table B: PCR reaction conditions for dideoxy sequencing analysis

| **PCR mastermix** | **KRas**  **exon 1** | **KRas**  **exon 2** | **KRas exon 3** | **BRaf**  **codon 600** | **PIK3CA exon 9** | **PIK3CA exon 20** |
| --- | --- | --- | --- | --- | --- | --- |
|  |  |  |  |  |  |  |
| **10x buffer** | 2.5 μl | 3 μl | 10 μl | 3 μl | 5 μl | 10 μl |
| **MgCl2** | 1.5 μl | 1.5 μl | 0 | 1.5 μl | 2.5 μl | 0 |
| **DMSO** | 0 | 0 | 3 μl | 0 | 0 | 1.5 μl |
| **dNTPs** | 2 μl | 2.4 μl | 1 μl | 2.4 μl | 2 μl | 2 μl |
| **Forward primer** | 1 μl | 0.3 μl | 0.5 μl | 0.3 μl | 1 μl | 1 μl |
| **Reverse primer** | 1 μl | 0.3 μl | 0.5 μl | 0.3 μl | 1 μl | 1 μl |
| **Taq -polymerase** | 0.2 μl | 0.2 μl | 0.2 μl | 0.2 μl | 0.2 μl | 0.5 μl |
| **H_2_O** | 17.8 μl | 22.2 μl | 34.8 μl | 22.2 μl | 38.3 μl | 33 μl |
|  |  |  |  |  |  |  |
| **Annealing temperature** | 57°C | 55°C | 54°C | 54°C | 58°C | 55°C |

PCR cycling conditions were 94°C for 5 minutes, followed by 35 cycles of 94°C for 30 seconds, (annealing temperature specified above) for 30 seconds, 72°C for 30 sec, followed by a final extension step of 72°C for 5 minutes.

Table C: PCR reaction conditions for pyrosequencing analysis
